# State Legislation, Regulations, and Hospital Guidelines for Newborn Screening for Critical Congenital Heart Defects — United States, 2011–2014

**Published:** 2015-06-19

**Authors:** Jill Glidewell, Richard S. Olney, Cynthia Hinton, Jim Pawelski, Marci Sontag, Thalia Wood, James E. Kucik, Rachel Daskalov, Jeff Hudson

**Affiliations:** 1 Division of Birth Defects and Developmental Disabilities, National Center on Birth Defects and Developmental Disabilities, CDC; 2 Division of Human Development and Disability, National Center on Birth Defects and Developmental Disabilities, CDC; 3 American Academy of Pediatrics, Elk Grove Village, Illinois; 4 University of Colorado—Denver; 5 Association of Public Health Laboratories, Silver Spring, Maryland; 6 Office of the Associate Director for Policy, Office of the Director, CDC

Critical congenital heart defects (CCHD) occur in approximately two of every 1,000 live births (1). Newborn screening provides an opportunity for reducing infant morbidity and mortality (2,3). In September 2011, the U.S. Department of Health and Human Services (HHS) Secretary endorsed the recommendation that critical congenital heart defects be added to the Recommended Uniform Screening Panel (RUSP) for all newborns (4). In 2014, CDC collaborated with the American Academy of Pediatrics (AAP) Division of State Government Affairs and the Newborn Screening Technical Assistance and Evaluation Program (NewSTEPs) to assess states’ actions for adopting newborn screening for CCHD. Forty-three states have taken action toward newborn screening for CCHD through legislation, regulations, or hospital guidelines. Among those 43, 32 (74%) are collecting or planning to collect CCHD screening data; however, the type of data collected by CCHD newborn screening programs varies by state. State mandates for newborn screening for CCHD will likely increase the number of newborns screened, allowing for the possibility of early identification and prevention of morbidity and mortality. Data collection at the state level is important for surveillance, monitoring of outcomes, and evaluation of state CCHD newborn screening programs.

Congenital heart defects occur in approximately eight of every 1,000 live births, one fourth of which are considered to be CCHD (1). CCHD are defined as those requiring surgery or catheterization before age 1 year. In the absence of early detection, infants with CCHD are at risk for serious complications or death within the first few days or weeks of life (1). Newborn screening for CCHD uses pulse oximetry, a noninvasive technology to measure blood oxygen saturation. Low oxygen saturation indicates hypoxemia, an early clinical sign of CCHD. Additional testing (e.g., repeat screening, echocardiogram) is needed following an abnormal pulse oximetry screen (1) to determine whether CCHD are present (or to determine the cause of the abnormal result). Thus, unlike most newborn screening conditions, screening for CCHD is not based on performing a blood test. In addition, hypoxemia detected by screening could indicate a medical problem, and requires immediate follow-up before discharge from the hospital.

When accompanied by early identification and treatment, newborn screening provides an opportunity to reduce infant morbidity and mortality (2,3). The Secretary’s Advisory Committee on Heritable Disorders in Newborns and Children has provided national guidelines and recommendations on newborn screening, known as the RUSP, and this panel is reviewed and endorsed by the HHS Secretary (3). As of March 2015, 32 conditions were included in the RUSP. States use the RUSP as guidance when considering adopting conditions for their own screening panels (3). State decisions might differ depending on method of screening required or the legislative authority of the newborn screening program. When states add conditions to their state-specific screening panels, they do so by state legislation, or rules and regulations (5). In 2010, the Secretary’s Advisory Committee on Heritable Disorders in Newborns and Children recommended adding CCHD to the RUSP for all newborns (4). In September 2011, the HHS Secretary endorsed the recommendation.

To assess states’ actions for adopting newborn screening for CCHD, CDC collaborated with the AAP Division of State Government Affairs and NewSTEPs. AAP obtained primary information through direct contact and partnership with AAP state chapters. AAP monitored state legislation by use of tracking software; regulations and hospital guidelines were researched on state websites.

NewSTEPs is a program of the Association of Public Health Laboratories in collaboration with the Colorado School of Public Health, funded through a cooperative agreement from the Health Resources and Services Administration (6). NewSTEPs maintains a data repository of state newborn screening program metrics and provides education and technical assistance to newborn screening programs. In January 2014, NewSTEPs distributed a survey on CCHD newborn screening adoption and data collection practices to state CCHD newborn screening programs. The survey requested the status of CCHD mandates and requirements for data collection. If data collection was required at the state level, additional information was requested on the type of data collected. All 50 states and the District of Columbia participated.

The survey findings indicated that 43 states have legislation, regulations, or hospital guidelines in place supporting CCHD newborn screening; 35 states have legislation, and 13 have regulations related to CCHD screening (Table). Among the 43, three states (Indiana, Maryland, and New Jersey) enacted legislation before the Secretary’s approval of adding CCHD to the RUSP in 2011 (Table). State adoption of CCHD screening peaked in 2013 with 25 states adopting screening (Figure 1).

The manner in which these 43 states developed universal screening varied substantially (Figure 2), and for some was a multistage process (Table). For example, California passed legislation requiring that CCHD screening be offered to parents of newborns. In 2013, Pennsylvania issued a regulation requiring reporting of results and diagnoses of screened newborns. However, the regulation did not mandate screening. In 2014, Pennsylvania enacted a law requiring screening. In 2012, Tennessee initially passed legislation that required the state’s genetic advisory committee to develop a program for addition of CCHD to its screening panel. In 2013, Tennessee added CCHD to its panel via regulation. In 2012, Virginia’s governor issued an executive order establishing a work group to develop a CCHD screening implementation plan, and legislation for mandatory screening was passed in 2014. In 2013, Massachusetts issued guidelines that recommended hospitals screen newborns and passed mandatory screening legislation in 2014. In 2014, Wisconsin enacted a law that allows the state department of health to add conditions to its state panel via regulation. Soon after enactment, regulations were issued adding CCHD to its panel.

Seven states and the District of Columbia support CCHD newborn screening as the standard of care with no mandate in place. Two states and the District of Columbia report that all hospitals are screening for CCHD (Table).

By December 2014, among the 50 states and the District of Columbia, data collection within each newborn screening program varied from no data collection to collection of all screening results for every newborn. Of the states that have implemented, or are planning to implement CCHD screening, 24 reported current data collection, 14 reported planning future data collection, and 13 reported no plans for data collection (Table). The types of data collection vary from aggregate data collection only, collection of pass/fail results on all newborns, oxygen saturation results on all newborns, oxygen saturation results on failed newborns only, or a combination of these (Table).

## Discussion

The increasing number of states mandating newborn screening for CCHD will likely increase the number of newborns screened, allowing for early identification and the potential for the prevention of morbidity and mortality. Most newborn screening conditions are tested through a heel stick test, with bloodspot analysis at public health or contracted laboratories. Screening for CCHD is a point-of-care test that occurs in hospitals before a newborn is discharged, with results entered into the medical record. Therefore, the role of public health is different than that for newborn bloodspot screening (7). This role might present challenges in data collection and surveillance for evaluating CCHD screening, because uniform reporting systems might not be established between public health programs, birthing centers, and hospitals (8). States have previously reported barriers to involvement with CCHD screening, such as the lack of legislative authority, staffing, funding, and informatics infrastructure (9). This report represents the first assessment of state legislative activities, requirements for collection of screening data, and progress made with screening activities, despite previously reported barriers.

State-level data collection is vital for surveillance, monitoring of outcomes, and evaluation of state CCHD newborn screening programs. Although all types of screening data can be valuable, individual-level data are important for surveillance and evaluation. Collecting data related to factors associated with false-positive and false-negative results could help refine the recommended CCHD screening algorithm and screening activities (7). As states evaluate the implementation of CCHD screening, they are encouraged to consider programmatic changes that would improve their screening program, such as the inclusion of individual-level data reporting.

Enactment of a state law or regulation does not translate into immediate and universal change in clinical practice. In addition to policy changes, the proper public health infrastructure, including infrastructure needs for data collection and reporting of CCHD screening results, is vital to ensure a successful CCHD newborn screening program.

**Summary**

What is already known on this topic?

Congenital heart defects occur in approximately eight in every 1,000 live births, one fourth of which are considered to be critical congenital heart defects (CCHD). Newborn screening using pulse oximetry can detect hypoxemia, a clinical sign of CCHD.

What is added by this report?

This report represents the first assessment of state’s actions to adopt newborn screening for CCHD and requirements for collection of CCHD screening data. Forty-three states have taken action toward newborn screening for CCHD through statute, regulations, or hospital guidelines. Among the 43 states, 32 (74%) are collecting or planning to collect CCHD screening data.

What are the implications for public health practice?

State mandates for newborn screening for CCHD might increase the number of newborns screened, allowing for early identification and prevention of morbidity and mortality. Data collection and reporting are essential to evaluate the effect of this public health program.

## Figures and Tables

**FIGURE 1:**
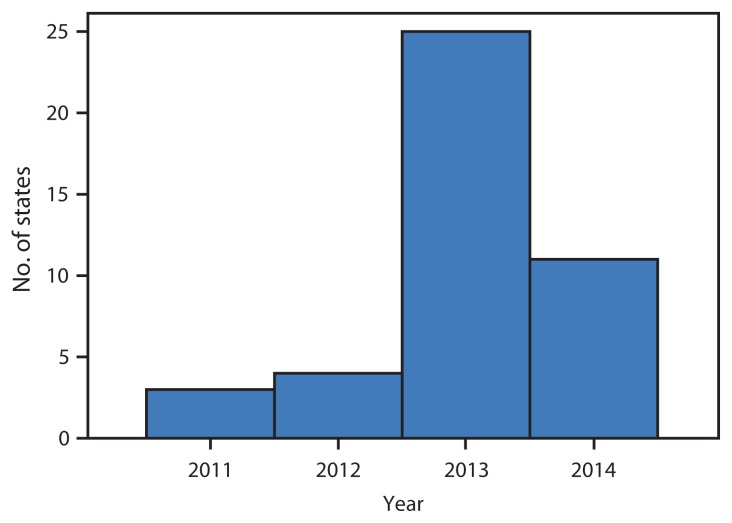
Number of states (N = 43) adopting legislation, regulation, or hospital guidelines for universal newborn screening for critical congenital heart defects, by year — United States, 2011–2014

**FIGURE 2:**
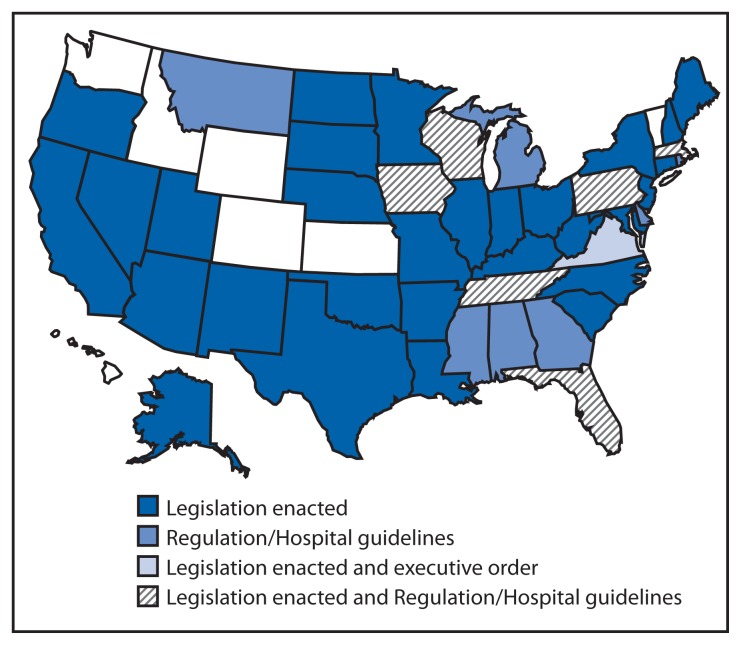
Actions taken by states to adopt newborn screening for critical congenital heart defects — United States, 2011–2014* * Actions taken as of December 2014.

**TABLE:** State approvals of legislation, regulation, and hospital guidelines for newborn screening for critical congenital heart defects (CCHD) — United States, 2011–2014

	Mechanism of current approval for CCHD screening						
			
State	Enacted date	Effective date	Legislation*	Regulation/Guidelines†	Screening supported as standard of care	Data collection system at state level	Type of data reported (current or proposed)
Alabama	May 2013	June 2013		X§		Planned	All oxygen saturations/times on all failed screens
Alaska	September 2013	January 2014 (January 2016 for providers who attend fewer than 20 births a year)	X§			Yes	Aggregate data
Arizona	April 2014	July 2015	X§			Planned	All oxygen saturations/times
Arkansas	April 2013	August 2013	X§			Planned	Pass/Fail on all newborns
California*	October 2012	July 2013	X (Screening is required to be offered)			Yes	Pass/Fail on all newborns All oxygen saturations/times on diagnosed cases
Colorado					X	Planned	All oxygen saturations/times
Connecticut	May 2012	January 2013	X§			No	
Delaware	May 2013	May 2013		X§		Yes	Pass/Fail on all newborns
District of Columbia					X¶	Yes	All oxygen saturations/times (one hospital)
Florida	October 2014	October 2014		X§		Yes	Final oxygen saturations/times
Georgia	May 2014	June 2014		X§		Planned	All oxygen saturations/times
Hawaii**					X	Planned	All oxygen saturations/times
Idaho					X	No	
Illinois	August 2013	August 2013	X§			No	
Indiana	May 2011	January 2012	X§			Yes	All oxygen saturations/times
Iowa (guidelines)	August 2012	August 2012	X§	X		No	
Iowa(legislation)	April 2013	July 2013					
Kansas					X	Yes	All oxygen saturations/times (four hospitals); Aggregate data (other hospitals)
Kentucky	March 2013	January 2014	X§			Yes	All oxygen saturations/times; Echocardiogram results††
Louisiana	June 2013	August 2013	X§			No	
Maine	July 2013	July 2013	X§			Planned	All oxygen saturations/times
Maryland	May 2011	July 2011	X§			Yes	Pass/Fail on all newborns; Option to enter all oxygen saturations/times
Massachusetts (guidelines)	May 2013	May 2013	X§	X		Yes	Aggregate data only
Massachusetts (legislation)	March 2014	January 2015					
Michigan	October 2013	April 2014		X§		Yes	All oxygen saturations/times; Echocardiogram results
Minnesota	May 2013	August 2013	X§			Yes	All oxygen saturations/times
Mississippi	October 2014	November 2014		X§		Planned	Aggregate data
Missouri	July 2013	January 2014	X§			Yes	Aggregate data; Plan to include newborn data with all oxygen saturations/times
Montana	June 2014	July 2014		X§		Planned	Pass/Fail on all newborns
Nebraska	June 2013	September 2013	X§			No	
Nevada	June 2013	July 2015	X§			Yes	Aggregate data only (hospitals participating in a pilot program)
New Hampshire	June 2012	August 2012	X§			No	
New Jersey	June 2011	September 2011	X§			Yes	Aggregate data; Plan to collect all oxygen saturations/times
New Mexico	March 2014	May 2014	X§			Planned	All oxygen saturations/times
New York	July 2013	January 2014	X§			No	
North Carolina	May 2013	May 2013	X§			Yes	Aggregate data
North Dakota	April 2013	August 2013	X§			No	
Ohio	June 2013	September 2013	X§			Planned	All oxygen saturations/times
Oklahoma	April 2013	July 2013	X§			Yes	Pass/Fail on all newborns
Oregon	June 2013	June 2013	X§			No	
Pennsylvania (regulation)†	December 2012 (regulation)	March 2013 (regulation)	X§	X		Yes	Aggregate data only; Oxygen saturations/time for confirmed cases only
Pennsylvania (legislation)	July 2014 (legislation)	September 2014 (legislation)					
Rhode Island	August 2014	July 2015		X§		Yes	Pass/Fail on newborns (some hospitals)
South Carolina	June 2013	June 2013	X§			No	
South Dakota	March 2013	July 2013	X§			No	
Tennessee (legislation)*	March 2012 (legislation)	January 2013 (legislation)	X	X§		Yes	Pass/Fail and date/time of screen on all newborns
Tennessee (regulation)†	May 2013 (regulation)	May 2013 (regulation)					
Texas	June 2013	September 2013	X§			Yes	All oxygen saturations on diagnosed cases only
Utah	March 2013	October 2014	X§			Yes	Pass/Fail on all newborns Planned: All oxygen saturations/times
Vermont					X¶	Planned	Aggregate data only on all newborns; Oxygen saturations/times on failed screens
Virginia (executive order)§§	June 2012	June 2012	X§			Planned	Oxygen saturations/times on failed screens
Virginia (legislation)	February 2014	July 2014					
Washington					X¶	No	
West Virginia	March 2012	June 2012	X§			Yes	Pass/Fail on all newborns
Wisconsin* (legislation)	March 2014 (legislation)	March 2014 (legislation)	X*	X§		Yes	Pass/Fail on all newborns; All oxygen saturations/times from some hospitals
Wisconsin (regulation)	June 2014 (regulation)	July 2014 (regulation)					
Wyoming					X	Planned	All oxygen saturations/times

* A total of 35 states have enacted legislation related to newborn screening for CCHD; 32 of those state laws require screening. California’s law requires the screen to be offered to parents of newborns before discharge. Tennessee’s law requires the state to develop a program for CCHD screening. Wisconsin’s law allows the state department of health to add conditions or diseases to the state’s newborn screening panel.

† A total of 13 states issued regulations or hospital guidelines related to newborn screening; 10 of those states issued regulations requiring screening. Iowa and Massachusetts issued guidelines to hospitals and birthing centers on screening, but the guidelines do not require screening. Pennsylvania issued a regulation requiring reporting of results and diagnoses of screened newborns, but the regulation does not require screening. Tennessee issued a regulation, after enacting legislation, adding CCHD to the state’s newborn screening panel.

§ Mandates CCHD screening of newborns.

¶ State reports that all hospitals are performing CCHD screening.

** Legislation in Hawaii to require screening failed in 2014.

†† Echocardiogram is the diagnostic test that follows a failed pulse oximetry screen.

§§ Virginia’s former governor issued a directive in 2012 that established a workgroup to develop a plan for implementing screening.
